# Chemical and Bandgap Engineering in Monolayer Hexagonal Boron Nitride

**DOI:** 10.1038/srep45584

**Published:** 2017-04-03

**Authors:** Kun Ba, Wei Jiang, Jingxin Cheng, Jingxian Bao, Ningning Xuan, Yangye Sun, Bing Liu, Aozhen Xie, Shiwei Wu, Zhengzong Sun

**Affiliations:** 1 The Department of Chemistry, Fudan University, Shanghai, 20433, China; 2 The Department of Physics and State Key Laboratory of Surface Physics, Fudan University, Shanghai, 20433, China; 3 Department of Chemistry and Shanghai Key Laboratory of Molecular Catalysis and Innovative Materials, Fudan University, Shanghai 200433, P.R. China

## Abstract

Monolayer hexagonal boron nitride (h-BN) possesses a wide bandgap of ~6 eV. Trimming down the bandgap is technically attractive, yet poses remarkable challenges in chemistry. One strategy is to topological reform the h-BN’s hexagonal structure, which involves defects or grain boundaries (GBs) engineering in the basal plane. The other way is to invite foreign atoms, such as carbon, to forge bizarre hybrid structures like hetero-junctions or semiconducting h-BNC materials. Here we successfully developed a general chemical method to synthesize these different h-BN derivatives, showcasing how the chemical structure can be manipulated with or without a graphene precursor, and the bandgap be tuned to ~2 eV, only one third of the pristine one’s.

Monolayer h-BN and graphene have similar atomic structures with a lattice mismatch of only 2%^1^,^2^ Therefore, the atomic layered h-BN is also called the “white graphene”. Graphene is a zero bandgap semimetal with spectacular electrical properties^3^, whereas h-BN is an insulator with a wide bandgap of ~6 eV^4^. Therefore, h-BN often serves as an atomic flat insulating substrate or a tunneling dielectric barrier in graphene and other 2D electronics^2^. Its special chemical properties and electronic structure also brought it with a wide range of technological applications, such as protective coating, deep ultraviolet emitter, and transparent membrane^5^,^6^. In addition, as an interesting optical material, h-BN shows natural hyperbolic behavior, which serves as an ideal platform for studying light–matter interactions and nanophotonic device engineering^7^,^8^.

To obtain high quality and pristine form of this material, significant efforts have been invested to devise chemical processes including mechanical cleavage^2^,^9^, liquid exfoliation^10^, radio frequency magnetron sputtering^11^, electron beam irradiation^12^, and chemical vapor deposition (CVD)^5^,^13^,^14^. For modern electronics purpose, high quality and uniform h-BN polycrystalline films and large sized single crystals are more preferred^5^,^11^,^13^,^14^ as lithographic suitable wafer-sized materials. Some recent work has displayed a preliminary success in growing triangular single crystals as large as ~130 μm^14^. To permanently alter h-BN’s band structure, it should structurally deviate from the pristine hexagonal form. The deviation includes chemically introduced vacancies, heteroatoms, 5- or 7-member rings^15^,^16^,^17^. Even for h-BN nanoribbons or quantum dots, their edges become dominated in shaping the band structures^18^,^19^. Uniaxial strain provides another way to break the crystal intrinsic symmetry^20^. Extrinsic applied electrical and magnetic fields could temporally bend some 2D materials’ band structures^21^. However, this needs to be testified in h-BN^22^. Recently, people reported exotic single-photon emission in h-BN, which can be attributed to one of the diverse engineered crystal structures^15^. In this work, with ammonia borane (BH_3_NH_3_) as the solid boron and nitrogen source, we not only produce the pristine and high-quality monolayer h-BN film and single crystals, but also new forms of hybrid structures using a PECVD method. These newborn structures could unfold different layers of this special 2D material and initiate brand new electrical applications other than just been a 2D dielectric.

## Pristine h-BN single crystals and films

**Figure 1:**
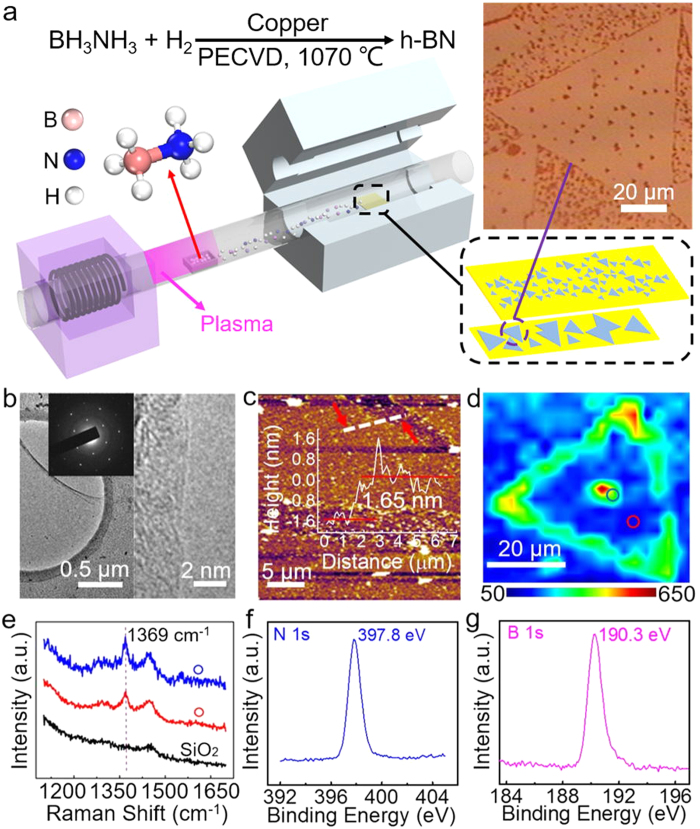
PECVD synthesis and microscopic properties of h-BN. (**a**) Schematic illustration of the growth procedure of h-BN. Inset: optical image of h-BN crystal on Cu foil. The triangle’s one side is 97 μm. (**b**) TEM image of monolayer h-BN. Inset: the corresponding SAED pattern. (**c**) AFM height image of monolayer h-BN domains transferred onto a 90 nm SiO_2_/Si substrate and the corresponding height profile along the white dashed line. (**d**,**e**) Raman mapping and Raman spectrum of h-BN single crystal, excited by a 532 nm wavelength laser for 20 s, and the laser power is 50 mW. (**f**,**g**) XPS spectra of B 1 s and N 1 s core level, respectively.

Figure 1a shows the procedure of growing pristine h-BN on Cu foil (see Methods and Supplementary Figure S1). 200 sccm high purity hydrogen (99.999%) was used as the carrier gas and excited into hydrogen plasma with a radio-frequency (RF) plasma generator (Zhongshan Kvmen Electronics Co., Ltd.). Also, the plasma generator’s frequency is 13.56 MHz, with its power set to be 60 W. Unlike traditional approaches where the BH_3_NH_3_ was heated up above 60 °C to generate vaporized B and N sources, hydrogen plasma can collide with the BH_3_NH_3_ white powder to produce various reactive species^23^. These fragments (B_x_N_y_H_z_^n±^) are the key feedstock in h-BN’s nucleation and growth. In order to decrease the nucleation density, another piece of Cu foil was placed on the growth substrate to prevent over nucleation and falling particulates (see Supplementary Figure S1). The grown crystals display a grain size ranging from 40 to 100 μm under different growth conditions. Most grains are uniform monolayers, except for small bilayer or multilayer seeds (see Supplementary Figure S2). Moreover, both the growth time and the distance between RF generator and the Cu foil could affect the size and quality of the final h-BN crystals (see Supplementary Figures S3, S4). Our experiments suggest that the optimized growth time for single crystal is ~30 min. In addition, longer growth time, such as 120 min, is favorable for the growth of full-coverage h-BN film (see Supplementary Figure S3). And the high quality h-BN crystals always grow at the center part of the 6 cm long Cu foil, which is ~75 cm away from the RF plasma generator. (see Supplementary Figure S4). After growth, h-BN was coated with poly(methyl methacrylate) (PMMA) and transferred onto SiO_2_/Si substrate for further characterizations, similar to previously reported graphene transfer^24^. Due to the weak optical contrast of h-BN on SiO_2_/Si substrate^25^, it is difficult to distinguish the triangle crystal of monolayer h-BN. However, we found that in our experiment, with a little PMMA impurity, the optical contrast could be enhanced sufficiently enough to distinguish these crystals from the background (see Supplementary Figure S5). The thin PMMA residuals on h-BN could be regarded as additional h-BN layers, which could enhance the optical contrast through both the light absorption and interference^25^,^26^.

Transmission electron microscopy (TEM) was used to characterize the crystal structure and thickness of the h-BN films. As shown in Fig. 1b, the TEM image clearly shows that the h-BN film was a monolayer^5^,^13^. The selected-area electron diffraction (SAED) pattern (inset in Fig. 1d) displays a distinctive hexagonal symmetry from the h-BN structure. In Fig. 1c, atomic force microscopy (AFM) reveals the 2D feature and the thickness of h-BN atop SiO_2_/Si substrate. The height profile (Fig. 1c and inset) indicates the h-BN film has a thickness ~1.65 nm, which is close to the 1 or 2 layers form of h-BN. Similar height profiles were collected from other samples, all of which were closed to 2 nm (see Supplementary Figure S6). It should be noted that the residual PMMA on h-BN and the surface roughness of SiO_2_/Si substrate could affect the AFM height measurements^5^,^27^. The roughness of the SiO_2_/Si substrate’s surface was ~0.3 nm measured with AFM (see Supplementary Figure S7). The PMMA residual and surface roughness of SiO_2_/Si introduced positive errors in measuring the thickness of h-BN, which makes it larger than the theoretic interlayer spacing ~0.33 nm. Other than the traditional ways to determine the thickness through TEM and AFM, X-ray photoelectron spectroscopy (XPS) might be another reliable tool to measure h-BN’s thickness^28^. However, to verify this method, we firstly need to grow standard and uniform mono-, bi-, tri-layer h-BN samples.

Raman spectroscopy is a sensitive technique to identify h-BN’s lattice vibration modes and distinguish it from other 2D materials. We performed Raman characterizations on our pristine h-BN transferred onto SiO_2_/Si substrate, using a 532 nm laser. Figure 1d is the Raman mapping of an h-BN triangular single crystal, with uniform E_2g_^29^ signals across the crystal except the nucleation center and edges. Figure 1e are typical Raman spectra from the h-BN single crystal (blue and red circle). Compared to the E_2g_ peak (~1366 cm^−1^) for bulk h-BN, the E_2g_ peak of the monolayer h-BN was blue-shifted to ~1369 cm^−1^ (see Supplementary Figure S8). No cubic boron nitride (c-BN) phonon modes, such as transverse optical phonon mode (~1057 cm^−1^) and longitudinal optical phonon mode (~1309 cm^−1^), were observed^30^,^31^. This suggests our PECVD method favors 2D h-BN crystals instead of c-BN.

To reveal its chemical environment and the elemental ratio of B and N, the as-grown monolayer h-BN samples were further probed with XPS. All XPS measurements were calibrated with the binding energy of the C 1 s peak at 284.5 eV (see Supplementary Figure S9). As presented in Fig. 1f,g, the binding energies of B 1 s and N 1 s are centered at 190.3 eV and 397.8 eV, respectively, which are similar to previous reported h-BN^1^,^2^. Both the B 1 s and the N 1 s spectra indicate that the chemical configuration for B and N atoms is solitary B-N bond, implying a pure and pristine phase of our h-BN films. The XPS elemental analysis confirms the ideal chemical stoichiometry of our h-BN crystals, with a B/N atomic ratio ~1:1^5^,^32^.

The Ultraviolet (UV)–visible absorption spectrum of monolayer h-BN film was applied to investigate the optical properties. Our h-BN films exhibit almost zero absorbance in the visible-light range and a sharp absorption in the UV region (~201 nm). The calculated optical bandgap (OBG) is ~6.17 eV, according to the formula for an indirect bandgap semiconductor (see Supplementary Figure S10).

## Grain boundaries in h-BN

Aside from the fixed three-fold symmetry and the alternative B-N atomic arrangement in the pristine form, the edge or GB were usually found to bear special optical or electrical properties^33^,^34^,^35^. Compared to the significant progress in mapping the GBs structure of graphene^34^,^35^ and MoS_2_^36^,^37^, the investigation of h-BN’s GBs is still in a premature stage. Theoretically, h-BN’s GBs, composed of an array of dislocations and non-hexagonal boron and nitrogen rings, could narrow the bandgap in some special orientations^38^,^39^. Owing to additional states along the boundary, the density functional theory (DFT) calculations predict the decrease of the bandgap as large as 38%^38^. Those theoretical predictions are the main driven force for our studies to make various h-BN’s GB structures and their related electronic properties.

**Figure 2:**
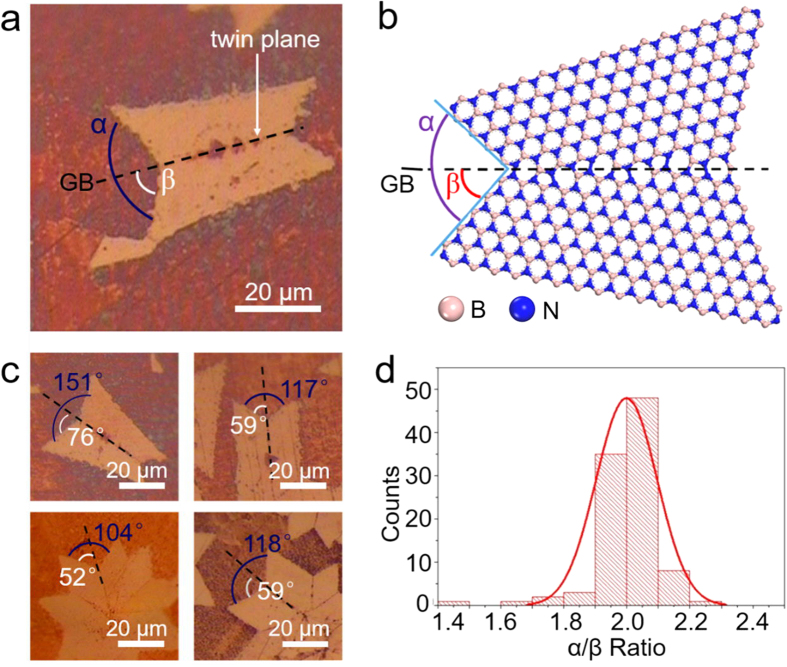
h-BN’s preferential grain boundary orientations. (**a**,**b**) Optical and atomic model illustrations of GB angle, the β and the crystal angle, the α. As a twin plane, the GB bisects the α. (**c**,**d**) Representative optical images showing that in different h-BN crystals, the GBs bisect the crystal angles and the statistic histogram of α/β ratios centers at 2.0 out of 100 h-BN crystals.

In our experiments, nucleation takes place in an uncontrolled fashion. Therefore, the as-grown h-BN single crystals are naturally merged and accompanied with GBs. GBs are interfaces between crystals growing with different orientations. Among all the GBs, twin GBs, mirrored across a twin plane (as Fig. 2a shows), are one of the simplest and most common ones^40^. GBs could appear straight at the microscopic level, but may not be true in the atomic level. We proposed several types of GBs, which could be formed and architected depending on the chemical bonding nature between the neighboring atomic configurations of B and N (see Supplementary Figure S11). In a typical atomic arrangement in Fig. 2b, the GB bisects the angle α, which locates between two joint crystals’ edges. It has been observed in some high resolution TEM (HRTEM) that the twin GB might consist of a series of 8/4 or 7/5 membered rings at atomic level^41^. According to the optical microscopy, the measured crystal angles (α) were mostly about twice of the GB angles (β). We measured 100 different h-BN GBs from different batch of samples, following the optimized growth parameters in the Supplementary Methods. The histogram in Fig. 2d shows the distribution of α/β ratio could be fitted into a Gaussian curve and centered at 2, suggesting the type of GBs is twin, and some of them could be coherent twin^39^,^40^. More optical measurements of the GBs were presented in Supplementary Figure S12. The GBs, forming when two crystals meet with each other, are more inclined to be twin boundaries than others. Although the width of the GBs are only a few angstroms, which is difficult to be observed without HRTEM^17^. Assisted with mild oxidation, direct observation on Cu surface of the GBs becomes possible by using optical microscope. Moreover, it is worth to be noted that, h-BN’s GBs could be observed through other non-linear optical method, such as the second harmonic generation (SHG)^42^.

Other than the GBs, atomic doping, alloying and hybridization proved to be another efficient pathway to alter material’s electronic structures. In spite of graphene and h-BN are completely different both atomically and electrically, their high lattice similarity allows their seamless inter-weaving into one piece of crystal^16^,^43^. Previous work has proved the concept that B, C and N could be atomically mixed together to form various semiconducting 2D structures with varying stoichiometry^43^ Theoretically, the electronic and magnetic properties of BN sheet could be modulated by embedding small graphene domains in it, while keeping its 2D planar geometry^44^. Recently, several attempts have been made to co-deposit graphene and h-BN to synthesize hybridized boron carbonitride (h-BNC)^16^. In addition, there are also several attempts to synthesize h-BN atomic layers by substitutions of carbon atoms with boron and nitride atoms^1^,^45^. The significance of the chemical and electrical tunabilities^16^,^43^,^45^ has motivated us to apply our PECVD method to synthesize other h-BN derivative materials.

## The growth of lateral heterojunctions from graphene single crystals

**Figure 3:**
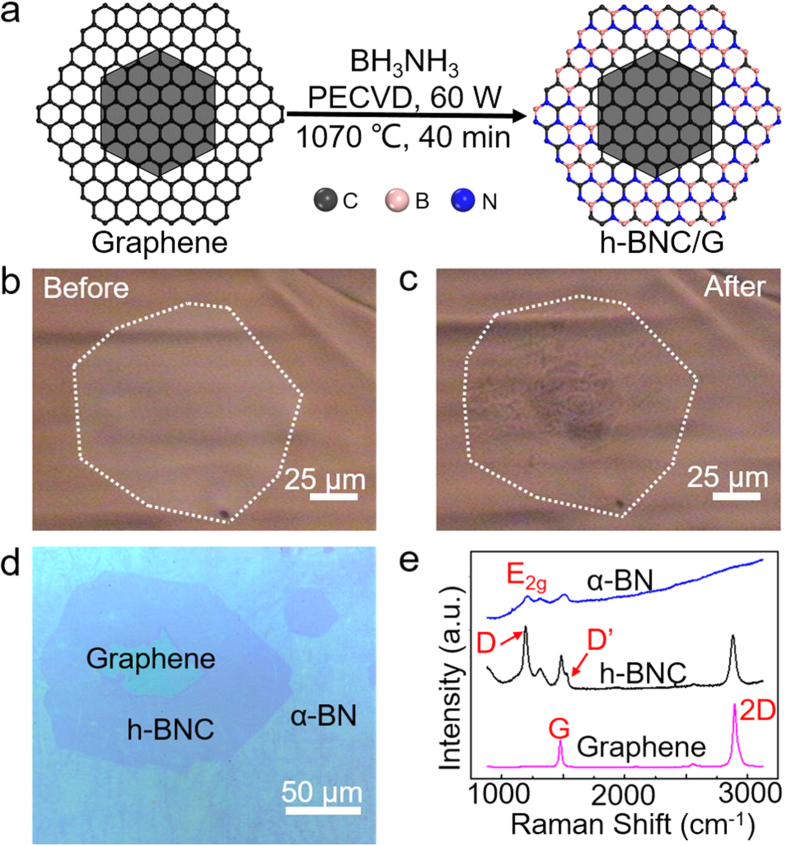
h-BNC/graphene (h-BNC/G) heterojunctions derived from graphene single crystals. (**a**) Schematic illustration of the synthesis procedure for h-BNC/G heterojunctions from a graphene single crystal with a multilayer center. (**b**,**c**) Optical images of the same graphene single crystal before and after the chemical conversion. (**d**) Optical image of h-BNC/G heterojunctions transferred on a 300 nm SiO_2_/Si substrate. (**e**) A corresponding room-temperature Raman spectrum of h-BNC/G heterojunctions exhibited in (**d**), excited with a 473 nm wavelength laser for 60 s, and the laser power is 3.3 mW.

Figure 3a presents an experimental scheme illustrating this conversion. During the growth, carbon atoms were substituted by boron and nitrogen atoms. The graphene single crystals were firstly synthesized using reported CVD method^34^. Then h-BN was grown following the same occupation of the graphene single crystals (see Supplementary Methods). Figure 3b,c show the optical images of the located graphene single crystal before and after the conversion, where the white dashed lines highlight the crystal’s shape. When it comes to the outline of converted h-BNC/G, there shows no optical difference from previous graphene crystal. After being transferred on the 300 nm SiO_2_/Si substrate (Fig. 3d), the layout of the h-BNC/G heterojunction’s composition became more obvious from their different optical contrasts. All three different regions, presumably the graphene, h-BNC and amorphous BN (α-BN), have shown their own distinct Raman signatures in Fig. 3e. It is noted that no D peak is observed for the centered graphene, suggesting the inner part might stay intact after being through the chemical conversion; and thereby the atomic conversion might initiate from the graphene crystal’s edge and slowly propagate into the center. For the h-BNC part, the Raman spectrum displays a sharp D peak at ~1358 cm^−1^. At the right shoulder of G band, a D’ peak appears at ~1623 cm^−1^. Besides, the h-BNC’s 2D peak intensity is diminishing compared to the pristine graphene^16^,^45^,^46^. Both the D and D’ bands are mainly attributed newly emerged BN domains or atomic doping inside the sp^2^ carbon plane, which breaks the original crystal symmetry. For α-BN, a strong E_2g_ peak is observed at ~1370 cm^−1^ and no obvious G or 2D peak can be detected (see Supplementary Figure S13), all three regions show distinguished photoluminescence spectra, indicating their lateral heterogeneous electronic structures (see Supplementary Figure S14).

## The growth of semiconducting h-BNC from graphene films

**Figure 4:**
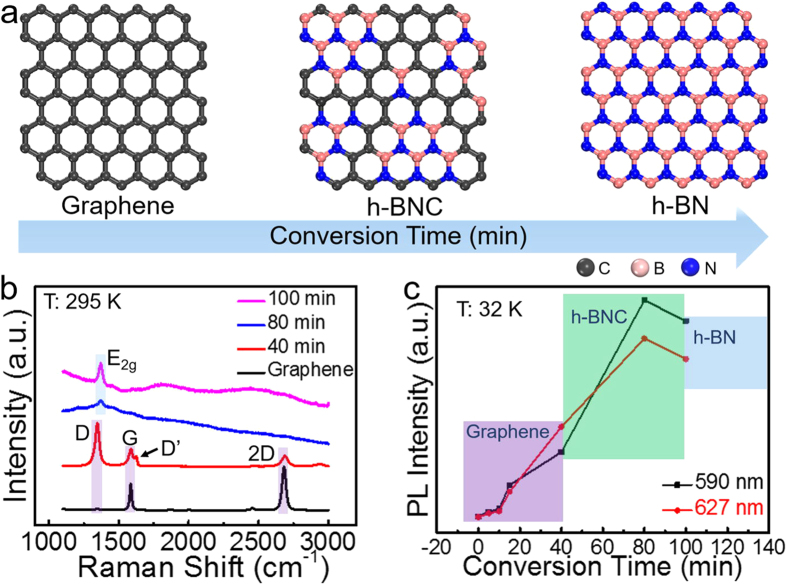
Hybridized h-BNC derived from graphene film under different conversion time. (**a**) Schematic illustration of the graphene, h-BNC and h-BN films. (**b**) Raman spectrum of hybridized h-BNC under different conversion time, excited by a 514 nm laser under 295 K for 10 s. The laser power is 4.8 mW. (**c**) Photoluminescence (PL) evolution of hybridized h-BNC under different conversion time. The excitation laser’s wavelength is 532 nm. The measurement was performed under 32 K for 10 s with a laser power of 20 μW.

As the graphene single crystal was successfully converted into an h-BNC crystal in Fig. 3, following an edge-to-center mechanism, and a whole graphene film could be divided into multi-domain graphene crystals, it becomes possible to transform a graphene film into h-BNC film at different stages with various conversion time. As illustrated in Fig. 4a, at first, the hydrogen can initiate etching and generate carbon vacancies, which plays the same role of the edges in the last single crystal reaction. Subsequently, the B and N atoms will fill in the vacancies where the carbon atoms have left. The h-BN domains grow larger and larger, and after a long conversion time, the graphene would eventually be converted into a complete h-BN film^16^.

Figure 4b shows the typical Raman spectra of pure graphene and hybridized h-BNC with different conversion time. The samples were assessed with different laser wavelengths and powers to acquire an optimized measurement conditions (see Supplementary Figure S15). The Raman on h-BNC favors the green laser and a non-saturated laser power as high as 4.8 mW. In Fig. 4b, we start with a pristine graphene film with no D peak observed, which indicates the absence of a detectable defect^46^. As the conversion develops, the D peak increases in the early 40 min and declines afterwards. Simultaneously, both the G and 2D peaks get diminished. These Raman characteristics are in accordance with the increasing levels of defects or functionalities in graphene^47^. Unlike the previous single crystal conversion, no distinct graphene’s Raman signatures (the G and 2D peaks) or h-BN’s (the E_2g_ peak) were found in those samples, therefore, eliminating the possible vertical heterojunction structures^43^. More likely, the hybrid structure may be constituted with alloys or mixed domains of h-BN, graphene and h-BNC. Especially when the conversion exceeds 15 min (see Supplementary Figure S16), the graphene’s D peak starts to slowly blueshift from 1354 cm^−1^ to the h-BN’s E_2g_ peak at 1368 cm^−1^.

We used PL spectroscopy to unveil the evolution of the band structures in the semiconducting h-BNC samples. Graphene and hybridized h-BNC sample were examined under low-temperature of 32 K (see Supplementary Figure S17) by a home-built PL set-up, using a 532 nm laser. The spectra exhibit two major PL peaks, ~590 nm (2.11 eV) and ~627 nm (1.98 eV), respectively. These two peaks follow the same trend in Fig. 4c, whose intensities reach to their zenith after ~80 min conversion. The narrowest bandgap in 80 min’s sample is ~2.11 eV, which is only about one third of h-BN’s original one. As the conversion time passes 100 min, more h-BN domains get converted, and the total PL intensity weakens. For all the h-BNC samples, the 80 min-converted one also shows the most homogeneous PL distribution map in Figure S17. The UV-visible absorption spectra in Figure S18 confirms the evolution from pristine graphene to h-BNC, and finally to h-BN. To further evaluate the electric properties of our h-BN and h-BNC films, we investigated the oxygen evolution reaction (OER) activities of these two materials. The polarization curves implied the distinguish differences between the insulating h-BN and semiconducting h-BNC (see Supplementary Figure S19).

The chemical pathways involve plasma activation, crystal nucleation, growth and multi-domain merging, graphene’s edge etching and atom substitution, which introduce great chemical and electronic diversities to the 2D materials. Pristine h-BN, GBs, heterojunctions and hybrid semiconducting h-BNC, all of these structures could be synthesized in one PECVD system without excessive modifications. Unambiguously we observed a dramatic bandgap narrowing in some of the h-BN’s derivatives, illuminating plenty of applications in 2D electronics or bandgap related electro-chemistries.

## Methods

The method of the materials synthesis and transfer are available in Supplementary Information of the paper.

### Characterizations

Optical microscope images were collected using the SK-2008H (Saike Digital) and Axio Imager A2 (Zeiss). Raman spectroscopy (HR Evolution and 800, Horiba) was used for sample analysis with 473 nm, 514 nm and 633 nm laser excitation. Low-temperature PL spectra was obtained by home-made PL set-up using a 532 nm wavelength laser. XPS (Axis Ultra DLD, Kratos) was used for elemental analysis of the samples (h-BN on Cu) using monochromatic Al Kα X-rays with the pass energy of 40 eV. The energy resolution of the XPS is ~1 eV and the take-off angle is 90°. The estimated probing depth for our XPS in current setup is 3 ~ 10 nm. Film thicknesses and topographical variations in the samples were measured using AFM (Asylum Cypher S and MTP-3D-Orign, Oxford). The TEM (JEM 2010, TEOL) was employed to study the morphology, crystal structure of the samples. UV-visible absorption spectroscopy was carried out with Shimadzu UV-3600 Plus spectrophotometer. The electrochemical work station (CHI 660E) was used for oxygen evolution reaction characterization.

## Additional Information

**How to cite this article:** Ba, K. *et al*. Chemical and Bandgap Engineering in Monolayer Hexagonal Boron Nitride. *Sci. Rep.* **7**, 45584; doi: 10.1038/srep45584 (2017).

**Publisher's note:** Springer Nature remains neutral with regard to jurisdictional claims in published maps and institutional affiliations.

## Supplementary Material

Supplementary Information

## References

[b1] Liu Z. . In-plane heterostructures of graphene and hexagonal boron nitride with controlled domain sizes. Nature Nanotech. 8, 119–124 (2013). 10.1038/nnano.2012.256 23353677

[b2] Dean C. R. . Boron nitride substrates for high-quality graphene electronics. Nature Nanotech. 5, 772–726 (2010). 10.1038/nnano.2010.172 20729834

[b3] Geim A. K. & Novoselov K. S. The rise of graphene. Nature Mater. 6, 183–191 (2007). 17330084 10.1038/nmat1849

[b4] Kubota Y., Watanabe K., Tsuda O. & Taniguchi T. Deep ultraviolet light-emitting hexagonal boron nitride synthesized at atmospheric pressure. Science 317, 932–934 (2007). 17702939 10.1126/science.1144216

[b5] Kim K. K. . Synthesis of monolayer hexagonal boron nitride on Cu foil using chemical vapor deposition. Nano Lett. 12, 161–166 (2012). 22111957 10.1021/nl203249a

[b6] Dai S. . Graphene on hexagonal boron nitride as a tunable hyperbolic metamaterial. Nature Nanotech. 14, 421–425 (2015). 10.1038/nnano.2015.131 26098228

[b7] Woessner A. . Highly confined low-loss plasmons in graphene–boron nitride heterostructures. Nature Mater. 14, 421–425 (2015). 25532073 10.1038/nmat4169

[b8] Caldwell J. D. . Sub-diffractional volume-confined polaritons in the natural hyperbolic material hexagonal boron nitride. Nature Commun. 5, 5221 (2014) 25323633 10.1038/ncomms6221

[b9] Lee C. . Frictional characteristics of atomically thin sheets. Science 328, 76–80 (2010). 20360104 10.1126/science.1184167

[b10] Coleman J. N. . Two-dimensional nanosheets produced by liquid exfoliation of layered materials. Science 331, 568–571 (2011). 21292974 10.1126/science.1194975

[b11] Sutter P., Lahiri J., Zahl P., Wang B. & Sutter E. . Scalable synthesis of uniform few-layer hexagonal boron nitride dielectric films. Nano Lett. 13, 276–281 (2013). 23244762 10.1021/nl304080y

[b12] Jin C., Lin F., Suenaga K. & Iijima S. Fabrication of a freestanding boron nitride single layer and its defect assignments. Phys. Rev. Lett. 102, 195505 (2009). 19518972 10.1103/PhysRevLett.102.195505

[b13] Gao Y. . Repeated and controlled growth of monolayer, bilayer and few-layer hexagonal boron nitride on Pt foils. ACS Nano 7, 5199–5206 (2013). 23663007 10.1021/nn4009356

[b14] Lu G. . Synthesis of large single-crystal hexagonal boron nitride grains on Cu-Ni alloy. Nature Commun. 6, 6160 (2015). 25606802 10.1038/ncomms7160

[b15] Tran. T. T. . Quantum emission from hexagonal boron nitride monolayers. Nature Nanotech. 11, 37–41 (2016). 10.1038/nnano.2015.242 26501751

[b16] Gong Y. . Direct chemical conversion of graphene to boron- and nitrogen- and carbon-containing atomic layers. Nature Commun. 5, 3193 (2014). 24458370 10.1038/ncomms4193

[b17] Gibb A. L. . Atomic resolution imaging of grain boundary defects in monolayer chemical vapor deposition-grown hexagonal boron nitride. J. Am. Chem. Soc. 135, 6758−6761 (2013). 23550733 10.1021/ja400637n

[b18] Zeng H. . “White graphenes”: Boron nitride nanoribbons via boron nitride nanotube unwrapping. Nano Lett. 10, 5049–5055 (2010). 21028887 10.1021/nl103251m

[b19] Li H. . Controllable synthesis of highly luminescent boron nitride quantum dots. Small 11, 6491–6499 (2015). 26574683 10.1002/smll.201501632

[b20] Ni Z. . Uniaxial strain on graphene: Raman spectroscopy study and band-gap opening. ACS Nano 2, 2301–2305 (2008). 19206396 10.1021/nn800459e

[b21] Sun Z., Martinez A. & Wang F. Optical modulators with 2D layered materials. Nature Photon. 10, 227–238 (2016).

[b22] Balu R., Zhong X., Pandey R. & Karna S. P. Effect of electric field on the band structure of graphene/boron nitride and boron nitride/boron nitride bilayers. Appl. Phys. Lett. 100, 052104 (2012).

[b23] Wolf G., Baumann J., Baitalow F. & Hoffmann F. P. Calorimetric process monitoring of thermal decomposition of B-N-H compounds. Thermochim. Acta 343, 19–25 (2000).

[b24] Li X. . Transfer of large-area graphene films for high-performance transparent conductive electrodes. Nano Lett. 9, 4359–4363 (2009). 19845330 10.1021/nl902623y

[b25] Orbachev R. V. . Hunting for monolayer boron nitride: optical and Raman signatures. Small 7, 465–468 (2011). 21360804 10.1002/smll.201001628

[b26] Kim K. K. . Synthesis and characterization of hexagonal boron nitride film as a dielectric layer for graphene devices. ACS Nano 6, 8583–8590 (2012). 22970651 10.1021/nn301675f

[b27] Bonaccurso E., Butt H.-J. & Craig V. S. J. Surface roughness and hydrodynamic boundary slip of a newtonian fluid in a completely wetting system. Phys. Rev. Lett. 90, 144501 (2003). 12731919 10.1103/PhysRevLett.90.144501

[b28] Seah M. P. & Spencer S. J. Ultrathin SiO_2_ on Si IV. Intensity measurement in XPS and deduced thickness linearity. Surf. Interface Anal. 35, 515–524 (2003).

[b29] Geick R. & Peery C. H. Normal modes in hexagonal boron nitride. Phys. Rev. 146, 543–547 (1966).

[b30] Werninghaus T., Hahn J., Richter F. & Zahn D. R. T. Raman spectroscopy investigation of size effects in cubic boron nitride. Appl. Phys. Lett. 70, 958–960 (1997).

[b31] Reich S. & Ferrari A. C. Resonant Raman scattering in cubic and hexagonal boron nitride. Phys. Rev. B 71, 205201 (2005).

[b32] Guo N. . Controllable growth of triangular hexagonal boron nitride domains on copper foils by an improved low-pressure chemical vapor deposition method. Nanotechnology 23, 1–6 (2012). 10.1088/0957-4484/23/41/415605 23011199

[b33] Suenaga K. & Koshino M. Atom-by-atom spectroscopy at graphene edge. Nature 468, 1088–1090 (2010). 21160475 10.1038/nature09664

[b34] Yu Q. . Control and characterization of individual grains and grain boundaries in graphene grown by chemical vapour deposition. Nature Mater. 10, 443–449 (2007). 10.1038/nmat3010 21552269

[b35] Huang P. Y. . Grain and grain boundaries in single layer graphene atomic patchwork quilts. Nature 469, 389–392 (2011). 21209615 10.1038/nature09718

[b36] Zande A. M. . Grains and grain boundaries in highly crystalline monolayer molybdenum disulphide. Nature Mater. 12, 554–561 (2013). 23644523 10.1038/nmat3633

[b37] Cheng J. . Kinetic nature of grain boundary formation in as-grown MoS_2_ monolayers. Adv. Mater. 27, 4069–4074 (2015). 26058724 10.1002/adma.201501354

[b38] Liu Y. . Dislocations and grain boundaries in two-dimensional boron nitride. ACS Nano 6, 7053–7058 (2012). 22780217 10.1021/nn302099q

[b39] Li Q. . Grain boundary structures and electronic properties of hexagonal boron nitride on Cu (111). Nano Lett. 15, 5804–5810 (2015). 26244850 10.1021/acs.nanolett.5b01852

[b40] Verhoeven J. D. Fundamentals of physical metallurgy (John Wiley & Sons, Inc., 1975).

[b41] Rong Y. . Controlled preferential oxidation of grain boundaries in monolayer tungsten disulfide for direct optical imaging. ACS Nano 9, 3695–3703 (2015). 25870912 10.1021/acsnano.5b00852

[b42] Kim C.-J. . Stacking order dependent second harmonic generation and topological defects in h-BN bilayers. Nano Lett. 13, 5660−5665 (2013). 24125021 10.1021/nl403328s

[b43] Ci L. . Atomic layers of hybridized boron nitride and graphene domains. Nature Mater. 9, 430–435 (2010). 20190771 10.1038/nmat2711

[b44] Kan M. . Tuning the band gap and magnetic properties of BN sheets impregnated with graphene flakes. Phys. Rev. B 84, 205412 (2011)

[b45] Sutter P., Cortes R., Lahiri J. & Sutter E. Interface formation in monolayer graphene-boron nitride heterostructures. Nano Lett. 12, 4869–4874 (2012). 22871166 10.1021/nl302398m

[b46] Ferrari A. C. . Raman spectrum of graphene and graphene layers. Phys. Rev. Lett. 97, 187401 (2006). 17155573 10.1103/PhysRevLett.97.187401

[b47] Englert J. M. . Scanning-Raman-microscopy for the statistical analysis of covalently functionalized graphene. ACS Nano 7, 5472–5482 (2013). 23668365 10.1021/nn401481h

