# Supplementary material for: Chemical and Bandgap Engineering in Monolayer Hexagonal Boron Nitride
Source: Sci Rep. 2017 Apr 3;7:45584. doi: 10.1038/srep45584 (PMC5377335; doi:10.1038/srep45584)
Supplement: Supplementary Information [file srep45584-s1.pdf]

Supplementary Information

Chemical and Bandgap Engineering in Monolayer Hexagonal Boron Nitride

Kun Ba[1,†] *, Wei Jiang*[1,†]*,Jingxin Cheng*[2]*, Jingxian Bao*[1]*, Ningning Xuan*[1]*,Yangye Sun*[1]*,*

Bing Liu[1]*, Aozhen Xie*[1]*, Shiwei Wu*[2]*, Zhengzong Sun*[1,3]*

[1]*The Department of Chemistry, Fudan University, Shanghai, 20433, China.* [2]*The Department of Physics and State Key Laboratory of Surface Physics, Fudan University, Shanghai, 20433, China.* [3]*Department of Chemistry and Shanghai Key Laboratory of Molecular Catalysis and Innovative Materials, Fudan University, Shanghai 200433, P. R. China.*

[†]*These authors contributed equally to this work.*

***email:** **zhengzong_sun@fudan.edu.cn**

Supplementary Methods

Hexagonal BN (h-BN) growth procedure. The h-BN was grown on the Cu substrate (Alfa Aesar, 99.8%, 25 μm) by using PECVD method. The Cu substrate was covered with a 2 cm × 6 cm Cu foil, placed in a 4-cm diameter quartz boat, and loaded into the PECVD furnace (Hefei Kejing Material Technology Co. Ltd. OTF-1200X). Subsequently, another quartz boat containing 10 mg ammonia borane (Alfa Aesar, 97%) was placed into the furnace. Then the Cu substrate was annealed at 1070 °C for 4 h under H_2 flow with a pressure of ~ 532 Torr. The distance from the plasma generator to

ammonia borane and copper was ~ 24 cm and 75 cm, respectively. The ammonia borane was decomposed under hydrogen plasma and brought to the Cu substrate for growth by H_2 flow. During the growth, the temperature of the furnace was kept at 1070 °C and the H_2 flow was maintained at 200 sccm. Meanwhile, the power of the frequency generator was set at 60 W. After growth for 30 min, the quartz boats containing Cu substrate were quickly cooled down to room temperature under the H_2 atmosphere [1-2].

Hybrid materials growth procedure. Hybrid materials growth were carried out in the PECVD. Graphene was grown on the Cu substrate and then loaded into the quartz tube, annealed at 1070 °C for 10 min under H_2 flow with the pressure to be about ~ 150 Torr. During the growth, the temperature was 1070 °C and the procedure lasted for 40 min with the RF power was 60 W.

Transfer Procedure. The PMMA solution (0.9 g/mL) was spin-coated on Cu substrate at 2500 rpm for 60 s and dried under 120 °C. Then the PMMA/h-BN/Cu was etched by using $FeCl_3$ solution (0.25 M, Sinopharm). The resulting PMMA/h-BN or PMMA/h-BNC membranes were rinsed with deionized water, and then transferred onto the SiO_2/Si substrate. After drying for overnight, the PMMA was removed with acetone and the sample was blow-dried with nitrogen gas [4].

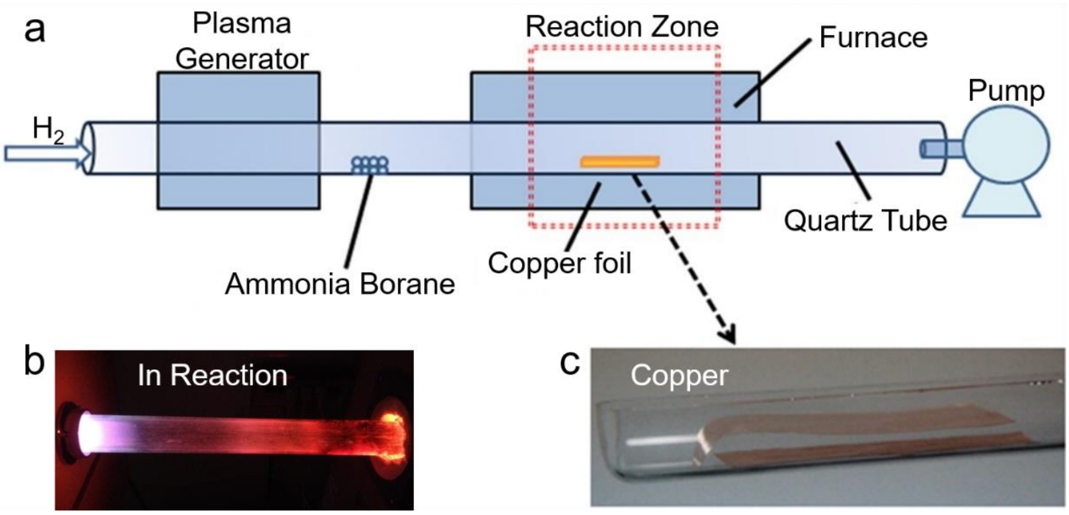

Figure S1 | (a) Schematic diagram of the PECVD setup for the growth of monolayer h-BN. (b, c) Illustration of the reaction procedure and the placement of Cu foils in the quartz tube, respectively.

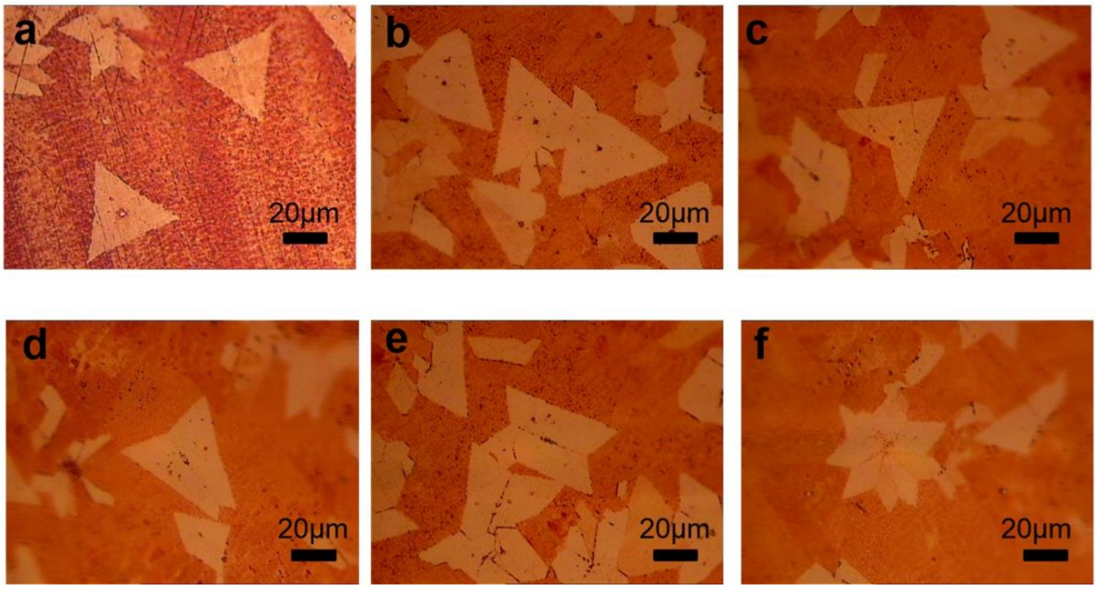

Figure S2 | Optical microscopy images of various PECVD-grown monolayer h-BN crystal shapes found in different growth on Cu substrates.

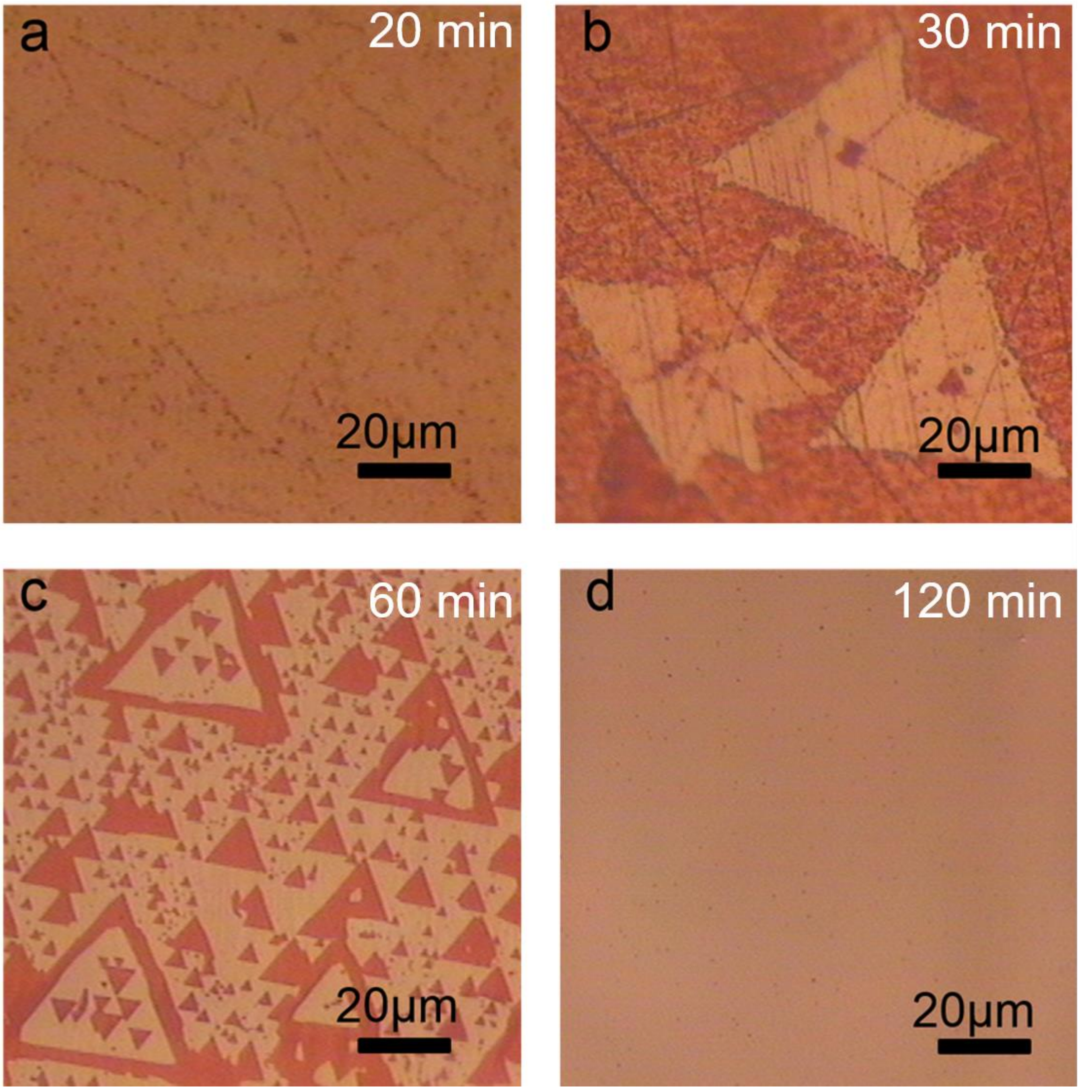

Figure S3 | Effect of growth time on the PECVD-grown monolayer h-BN. Optical microscopy images of h-BN grown for **(a)** 20 min, **(b)** 30 min, **(c)** 60 min, and **(d)** 120 min, while the other experiment conditions remained the same. Comparing the growth result between 30 min and 20 min, it appears that the initial flakes are increased both in size and density for longer growth times. The h-BN flakes growing across Cu grain boundaries are also observed frequently. When comes to 60 min, the density of the nucleus is much more than 30 min, but the grain size is decreased. Moreover, after growth for 120 min, the surface of Cu substrate is mostly covered by continuous h-BN film. The exact reason why longer or shorter growth time is not good for the single

crystal growth is not well understood. Probably, it is because that the reactive precursors were activated by the H plasma, which can also etch the h-BN crystals. In the early stage of the growth (0-30 min), it helps to etch away the small crystals and feeds the large crystals (the Ostwald ripening). While in the late stage (~ 60 min), the large crystals were also etched from its inside and broken into smaller triangular crystals as shown in Figure S3c. Finally, all small crystals merge into one continuous film as the growth proceeds to 120 min.

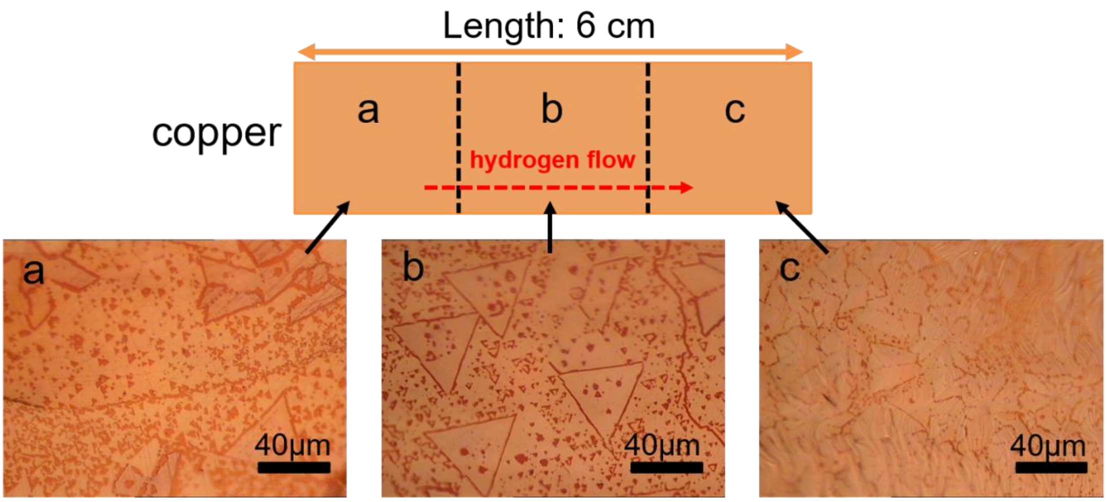

Figure S4 | Effect of the distance of the frequency generator to the Cu substrate on the PECVD-grown monolayer h-BN. (a) is the forepart of the Cu substrate, as it is close to the frequency generator, the vapor phase B and N sources may not stable, the monolayer h-BN crystals are not regular. **(b)** is the middle part, the crystals can be regular and the size is larger. **(c)** is the backend, it mostly be film and the crystals are still not regular. So we always chose the middle part of the Cu substrate grown on h-BN for further experimental.

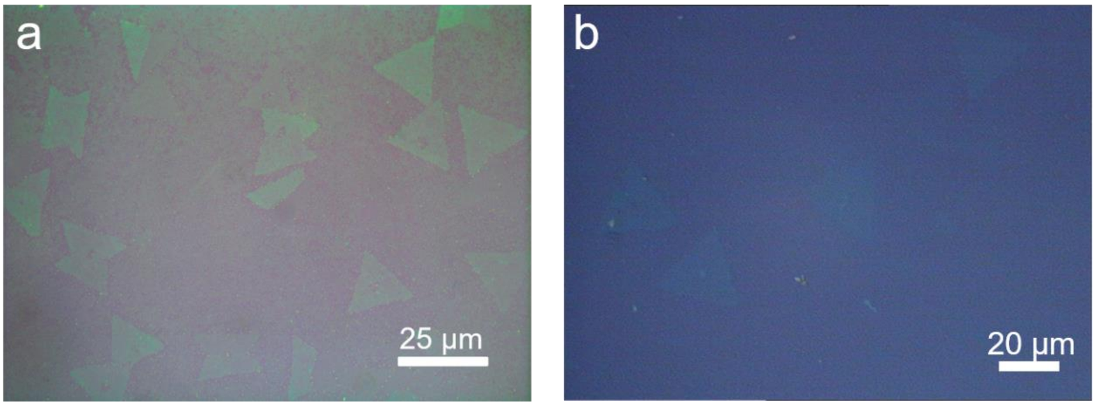

Figure S5 | Optical microscopy image of h-BN single crystals transferred on 300 nm SiO_2/Si substrate. Compared with b, there is more PMMA residual on the h-BN film in a, which leads to a higher optical contrast.

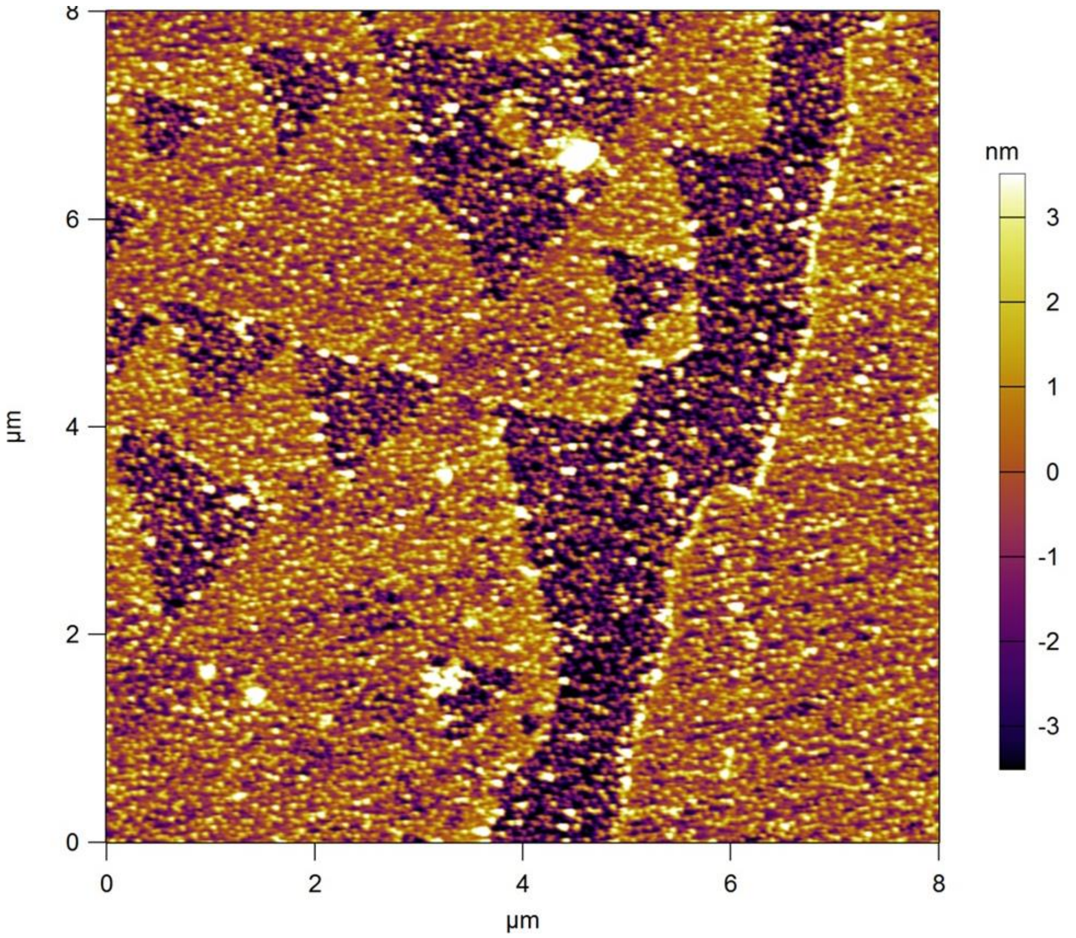

Figure S6 | AFM image of PECVD-grown monolayer h-BN domains transferred on 90 nm SiO_2/Si substrate. The thickness of the h-BN film is ~ 2 nm.

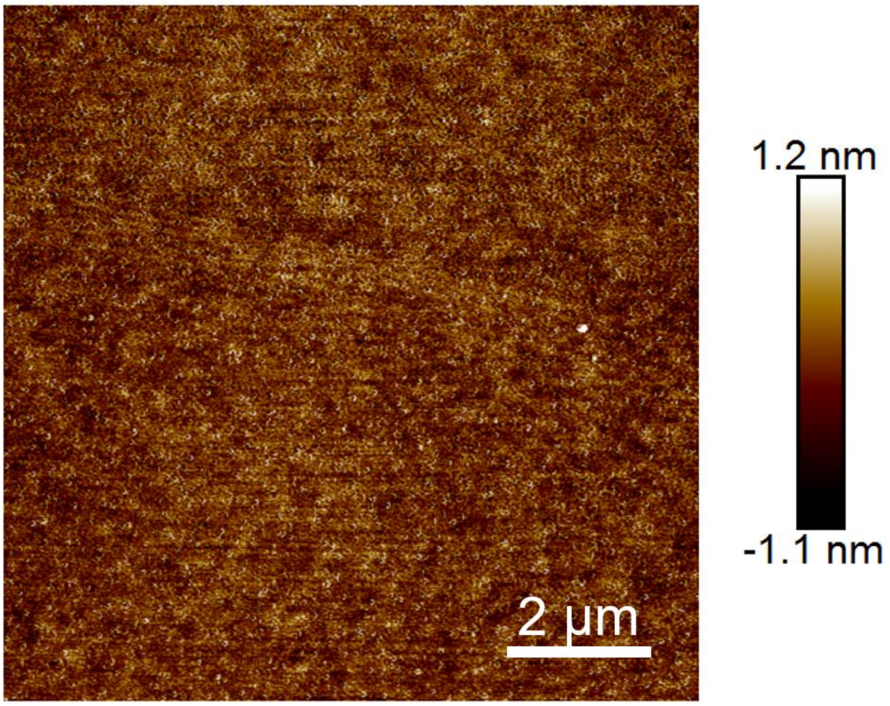

Figure S7 | AFM image of the surface of the as-used 90 nm SiO_2/Si substrate. The roughness of the substrate is ~ 0.3 nm.

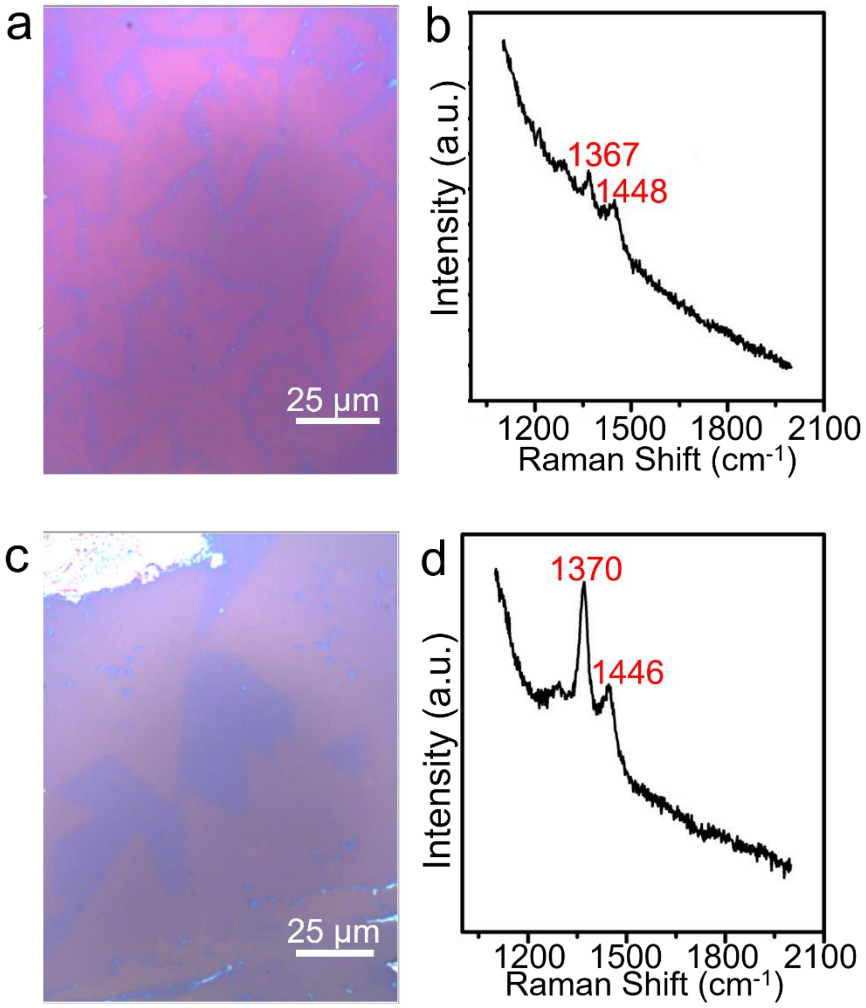

Figure S8 | Optical microscopy images (a, c) and the corresponding room-temperature Raman spectrum (b, d) of as grown h-BN, excited by 532 nm laser.

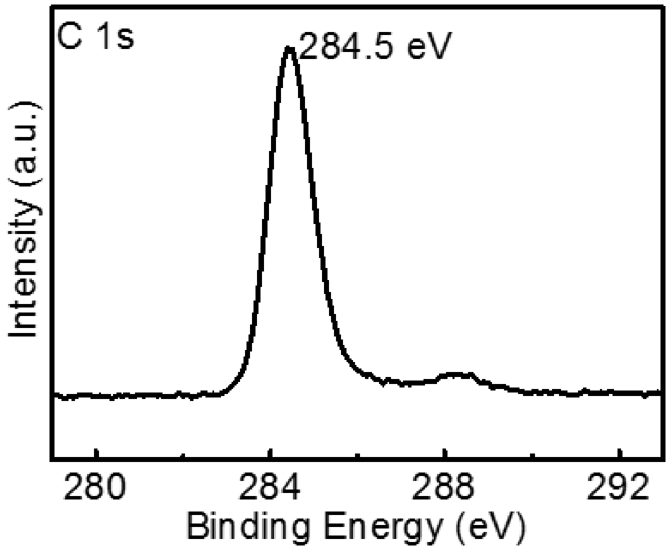

Figure S9 | XPS spectra of C 1s peak. For XPS calibration, we used the binding energy of the C 1s at 284.5 eV.

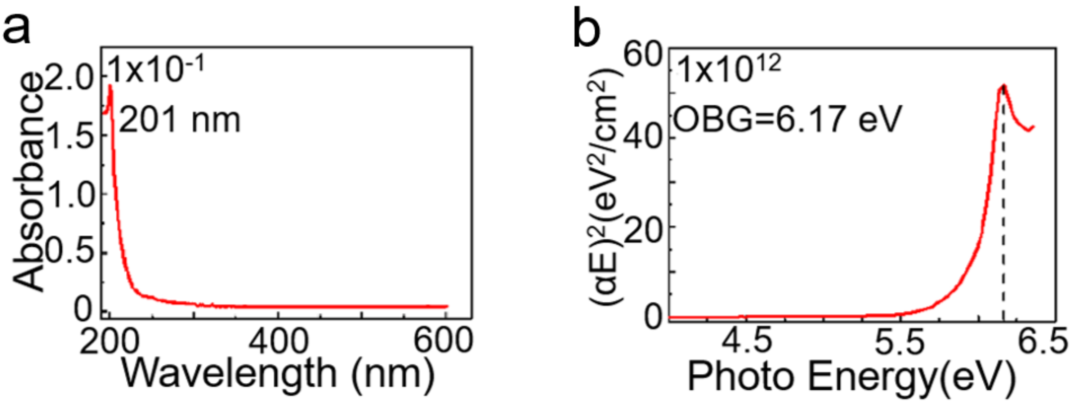

Figure S10 | (a) Ultraviolet-visible absorption spectrum of h-BN films taken at room temperature. (b) Tauc plot of a transferred h-BN film to determine the OBG.

As Figure S10a shows, the h-BN film is highly transparent from visible to UV wavelength and the absorption spectrum displays one sharp absorption peak at 201 nm. The optical band gap can be calculated based on $\alpha=C(E–E_g)^{0.5}/E =A/d$, where α is the absorption coefficient, C is a constant, E is the photon energy, E_g is the optical band gap, A is the optical absorbance, and d is the thickness of the h-BN film. To computer the optical band gap, we plot $(\alpha E)^2$ versus E, as shown in figure S7 b, and extrapolate the straight line of the energy dispersion curve to the E axis to obtain the value of E_g, which was found to be ~ 6.17 eV in this work. Previous theoretical calculations of band structures for a single layer of h-BN anticipated that equivalent bands did not cross each other and a 6eV band gap was the result [1-2].

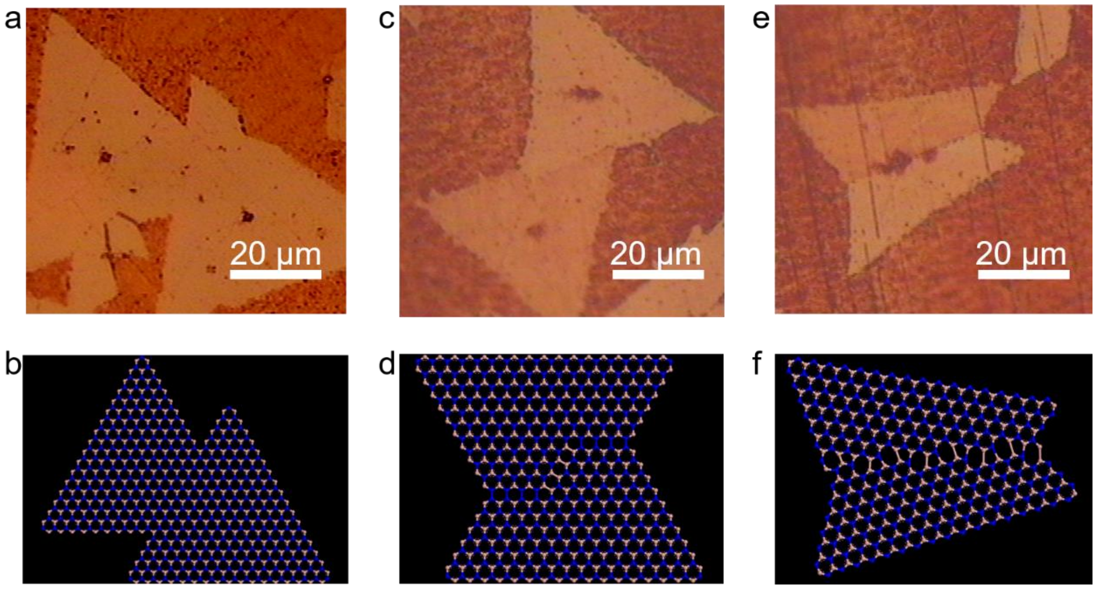

Figure S11 | Formation of PECVD-grown monolayer h-BN GB structure. (a, b) Optical microscopy images and schematic atomic showing BN/BN structure in as-synthesized h-BN joined domains. **(c, d)** Optical microscopy images and schematic atomic showing BN/NB structure in as-synthesized h-BN joined domains. (e, f) Optical microscopy images and schematic atomic showing dislocations in as-synthesized h-BN joined domains [5]. The creation of GBs occurs when one single crystal merges with another single crystal of different orientation, while the GBs may appear different structure at the microscopic level.

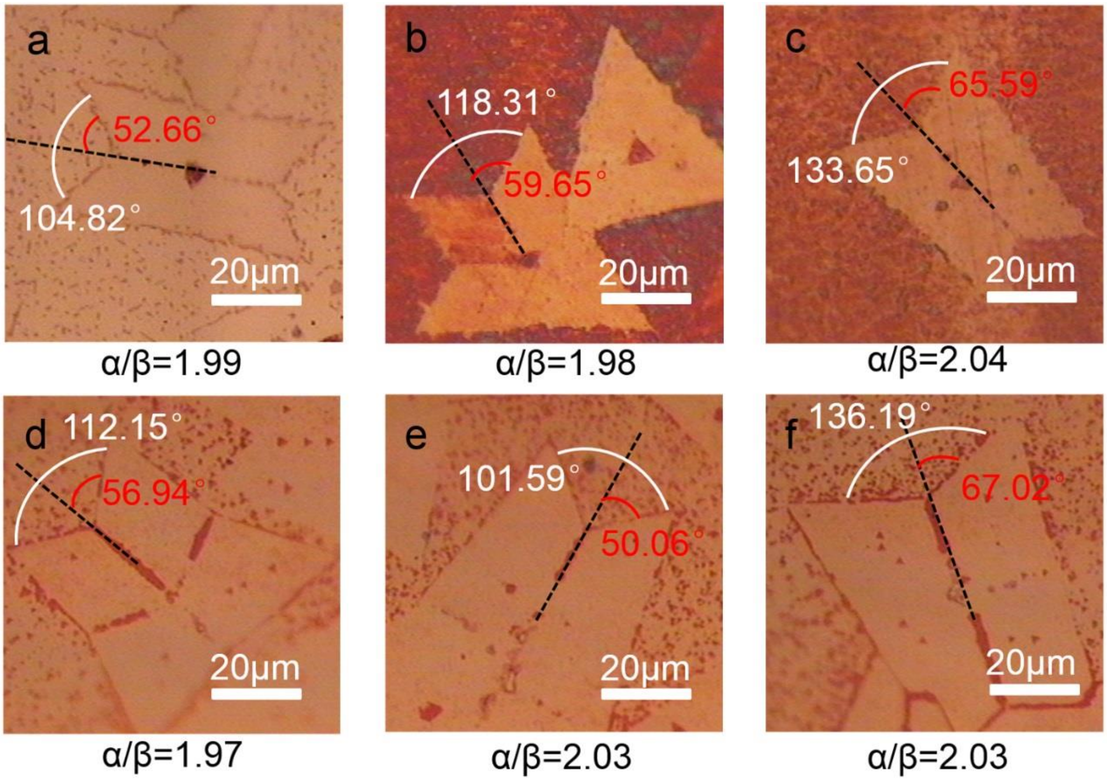

Figure S12 | Optical microscopy images to measure the domain angle (α, white) and GB angles (β, red) and the α to β ratios in monolayer h-BN joined domains.

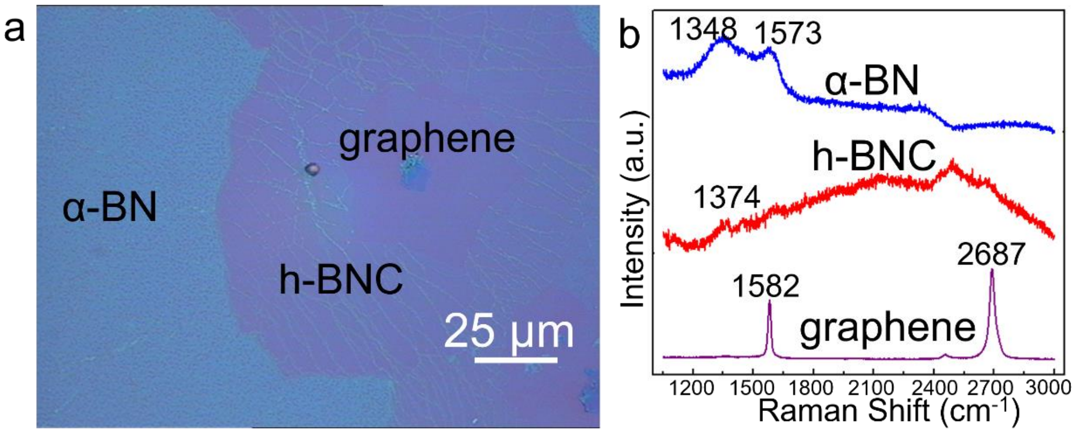

Figure S13 | Optical microscopy image and a corresponding room-temperature Raman spectra of as-grown h-BNC/G heterostructure, excited by 532 nm wavelength laser.

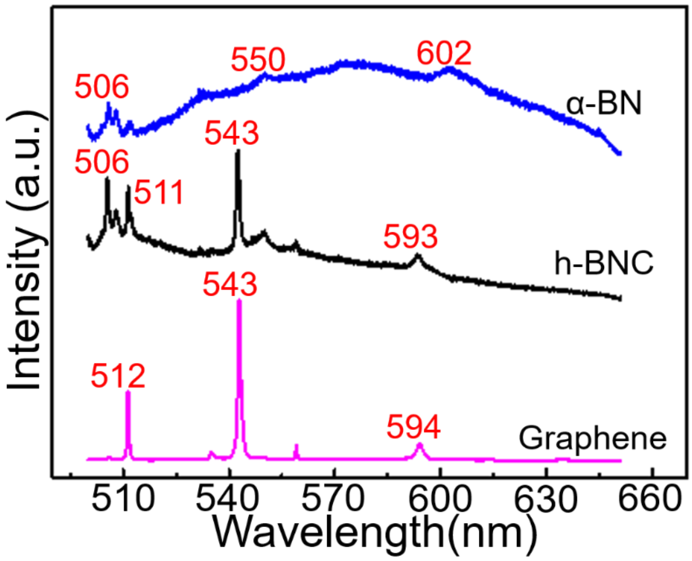

Figure S14 | Room-temperature photoluminescence spectrum of h-BNC/G heterostructure, excited by 473 nm wavelength laser.

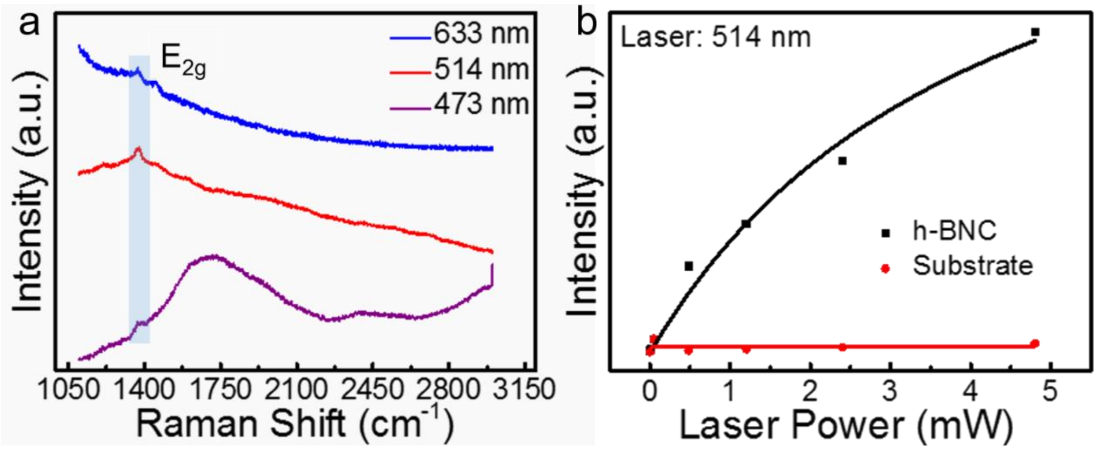

Figure S15 | (a) Room-temperature Raman spectra of 80 min converted hybridized h-BNC, excited by different wavelength lasers for 10 s. (b) Optimizing the laser power of 514 nm for the room-temperature Raman spectra of h-BNC (80 min). From Figure S15 a, we can know that, when the hybridized h-BN was excited by 514 nm wavelength laser, the E_{2g} peak could be sharper and clearer than the other two wavelength laser. As Figure S15 b shows, dependence of Raman intensity on the laser power when the wave number is 1369 cm^{-1}, we tried to confirm the optimized laser power of hybridized h-BN's Raman spectra obtained. As the black curve shows, along with the increase of laser power, the Raman intensity of the hybridized h-BN could be gradually stronger. And the Raman intensity is no trend toward the saturation. We also absorbed the Raman intensity of substrate. From the red curve we could know that the substrate has no influence on the Raman intensity of hybrid h-BN. Therefore, the Raman spectra were excited by 514 nm wavelength laser and the laser power is 4.8 mW in Figure 4b.

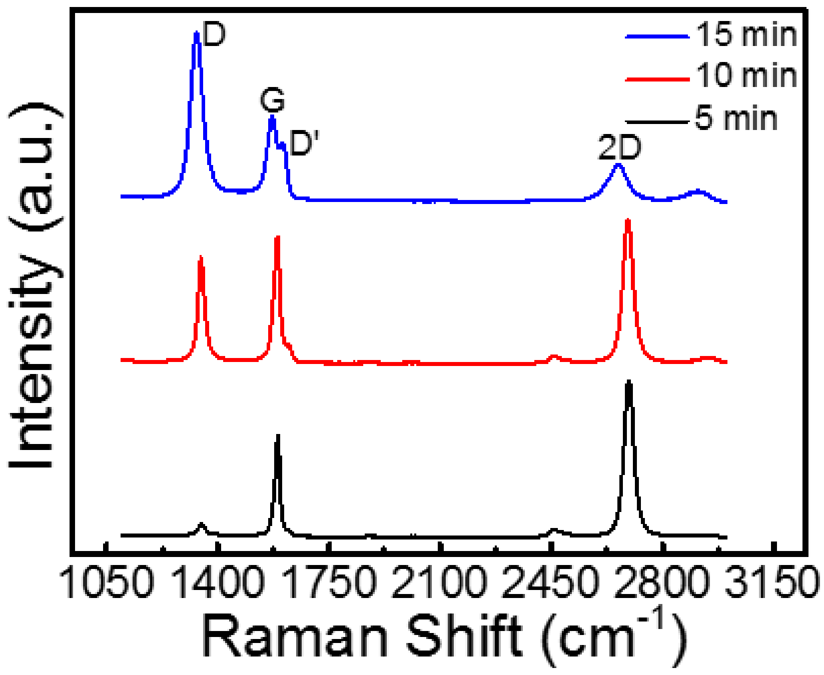

Figure S16 | Room-temperature Raman spectrum of hybridized h-BNC under different conversion time, excited by a 514 nm wavelength laser for 10 s, and the laser power is 4.8 mW. The typical Raman spectra of hybridized BN under different conversion time. During the converted procedure, the D peak could increase along with the conversion time from 5 min to 15 min. Simultaneously, the intensity of G and 2D peak decrease. As Figure S16 shows, when the conversion time is 15 min, at the right shoulder of G band, the D’ peak is also observed. Both the D and D’ bands could be attributed newly emerged BN domains inside the sp^2 carbon network.

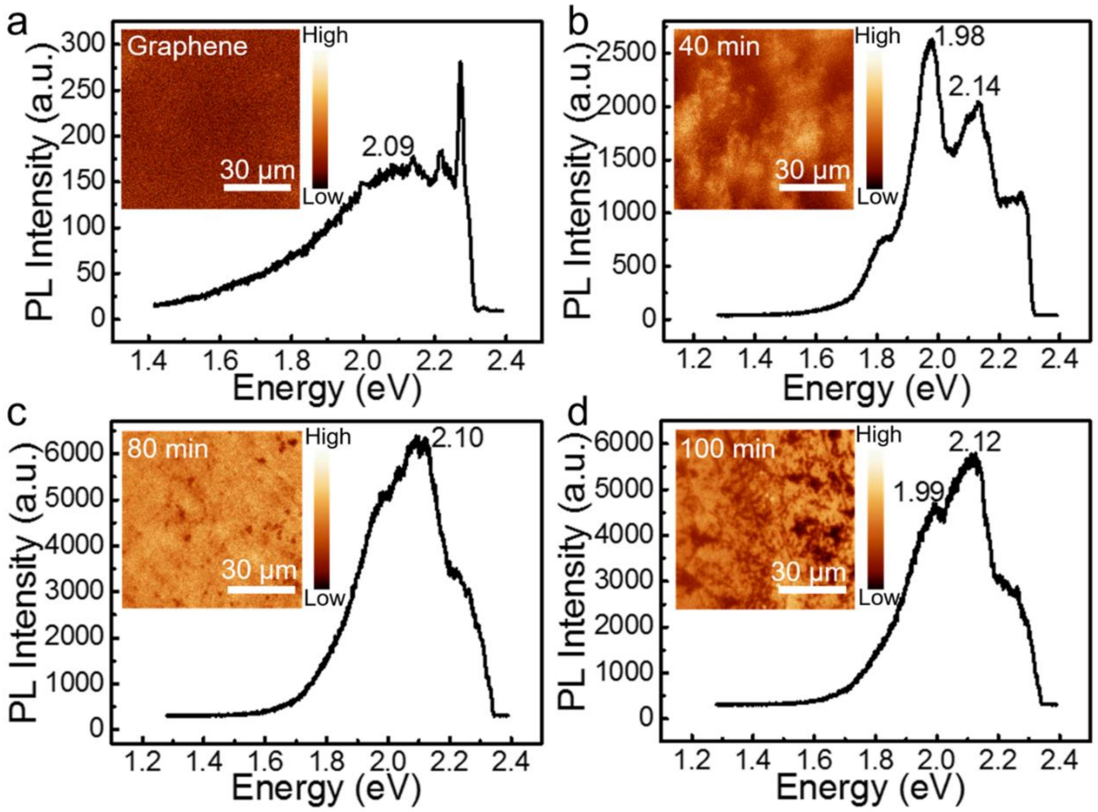

Figure S17 | PL spectra of graphene and hybridized h-BNC, excited by a 532 nm wavelength laser under 32 K for 10 s, and the laser power is 20 μW. Insets: PL intensity mapping. Before the growth time is 80 min, along with the increase of the conversion time, the PL intensity could be stronger. During this process, pristine graphene gradually converted into hybridized h-BN. As graphene is a zero bandgap semiconductor, there is no PL phenomenon. However, previous studies have found that the bandgap of the hybridized h-BN was higher than graphene, but lower than h-BN [7-8], so the PL intensity could be gradually stronger with the increase of the conversion time. What's more, when the conversion time increased to 100 min, the ingredient of h-BN would further increase, and then the PL intensity could weaken [9].

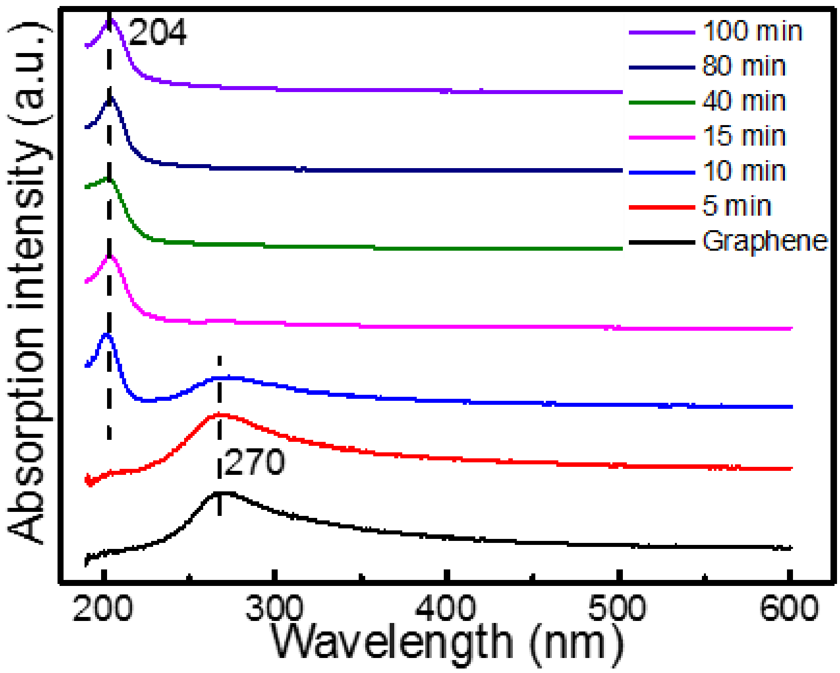

Figure S18 | UV-visible absorption spectra of pristine graphene and hybridized h-BNC. Owing to optically induced transitions, the UV–visible absorption spectra were taken to investigate the optical energy gap of graphene and hybridized BN. As Figure S18 demonstrates, the absorption peak of pure graphene is very weak in the range of 190-450 nm. When the growth time is longer than 40 min, the absorption spectra show one absorption peak at 204 nm, corresponding to an OBG of 6.09 eV determined from previous Tauc's formulation (see Supplementary Figure S10) [1]. Importantly, there appear two obvious absorption peaks in the absorption spectrum at 202 nm and 270 nm when the growth time is 10 min, which are very close to pure h-BN and graphene. The facts we observe two different absorption results indicate that graphene could gradually convert into hybridize h-BN. Moreover, both h-BN and graphene domains could coexist in the hybridized BN.

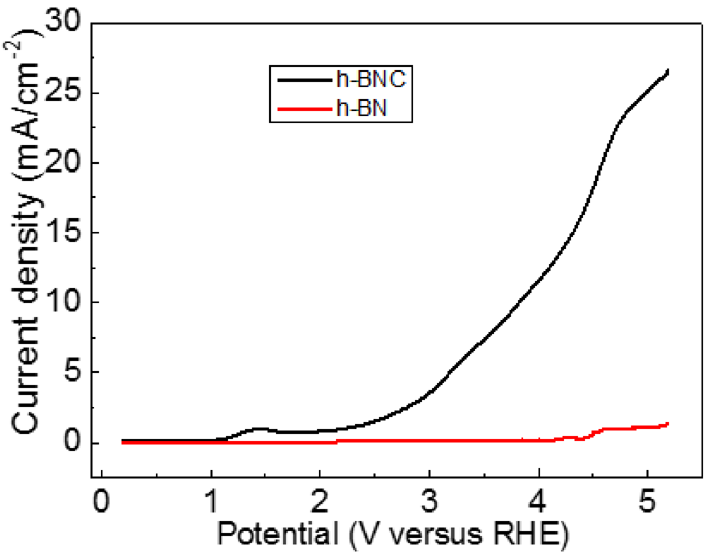

Figure S19 | Polarization curves of h-BN and h-BNC samples as indicated. h-BN and h-BNC film electrodes were fabricated and directly applied in standard three-electrode system for oxygen evolution reaction (OER) characterization in 0.5 M H_2SO_4. Polarization curves here demonstrate the substantial catalytic enhancement of h-BNC though high overpotential is needed. It implies the possible transition from insulator h-BN to semiconductor h-BNC.

Reference

1. Kim, K. K. *et al.* Synthesis of monolayer hexagonal boron nitride on Cu foil using chemical vapor deposition. *Nano Lett.* **12**, 161-166 (2012).
2. Song, L. *et al.* Large scale growth and characterization of atomic hexagonal boron nitride layers. *Nano Lett.* **10**, 3209–3215 (2010).
3. Kim, G. *et al*. Growth of high-crystalline, single-layer hexagonal boron nitride on recyclable platinum foil. *Nano Lett.* **13**, 1834-1839 (2013).
4. Suk, J. W. *et al*. Transfer of CVD-grown monolayer graphene onto arbitrary substrates. *ACS Nano* **5**, 6916-6924 (2011).
5. Kim, C-J *et al.* Stacking Order Dependent Second Harmonic Generation and Topological Defects in h‑BN Bilayers. *Nano Lett.* **13**, 5660−5665 (2013).
6. Ci, L. *et al.* Atomic layers of hybridized boron nitride and graphene domains. *Nature Mater.* **9**, 430-435 (2010).
7. Gong, Y. *et al.* Direct chemical conversion of graphene to boron and nitrogen- and carbon-containing atomic layers. *Nature Commun.* **5**, 3193 (2014).
8. Liu, Z. *et al.* In-plane heterostructures of graphene and hexagonal boron nitride with controlled domain sizes. *Nature Nanotech.* **8**, 119-124 (2013).
9. Horcas, I. *et al*. WSXM: A software for scanning probe microscopy and a tool for nanotechnology. *Rev. Sci. Instrum.* **78**, 013705 (2007).
